# Realistic 3D-Printed Lumbar Spine Model for Non-cadaveric Surgical Training: A Proof of Concept Study

**DOI:** 10.7759/cureus.81297

**Published:** 2025-03-27

**Authors:** Todor G Bogdanov, Hristo R Tsonev, Dimo A Yankov, Rene D Mileva-Popova, Dilyan Ferdinandov

**Affiliations:** 1 Medical Physics and Biophysics, Medical University of Sofia, Sofia, BGR; 2 Neurosurgery, St. Ivan Rilski University Hospital, Sofia, BGR; 3 Physiology and Pathophysiology, Medical University of Sofia, Sofia, BGR; 4 Neurosurgery, Medical University of Sofia, Sofia, BGR

**Keywords:** 3d-printed spine model, cost-effective physical models, educational model, lumbar spine, non-cadaveric surgical training

## Abstract

Surgical simulation plays a crucial role in modern neurosurgical training, allowing surgeons to develop and refine their skills in a controlled and risk-free environment. Traditional methods, such as cadaveric dissections, and virtual reality (VR) simulations more recently have their advantages and limitations. While cadaveric models offer high anatomical accuracy, they are expensive, difficult to access, and non-reusable. VR simulations provide customizable training experiences but lack the realistic haptic feedback necessary for hands-on procedures.

With advancements in 3D printing technology, anatomically accurate and cost-effective physical models have emerged as a viable alternative for surgical training. This study aims to develop and validate a realistic 3D-printed lumbar spine model for non-cadaveric surgical education. The proposed model replicates the anatomical and biomechanical properties of the L1-S1 segment and is produced using fused deposition modeling (FDM) 3D printing technology with polylactic acid (PLA) vertebrae, PolyFlex TPU95 intervertebral discs, and an elastic TPU (thermoplastic polyurethane) rod to mimic physiological movement.

The model is based on DICOM imaging data from a CT scan of a patient’s spine, optimized for biomechanical resistance and realistic pedicle screw placement training. It was tested in hands-on neurosurgical workshops at St. Ivan Rilski University Hospital in Sofia. Post-training X-ray analysis confirmed the accuracy of screw positioning and the anatomical fidelity of the model.

The results demonstrate that this 3D-printed lumbar spine model provides an accessible, customizable, and reliable training tool for spine surgery. Future improvements may include multi-material printing, augmented reality (AR) integration, and adaptations for pathological conditions.

## Introduction

Surgical simulation is crucial in modern neurosurgical training, enabling surgeons to develop and refine their skills in a controlled and risk-free environment [1]. Traditional methods, such as cadaveric dissections and, more recently, virtual reality (VR) simulations, each present their own advantages and limitations. While cadaveric models provide high anatomical fidelity [2], they are expensive, difficult to access, and lack reusability. VR-based simulations, on the other hand, offer a safe and customizable training experience but often fail to replicate the haptic feedback required for hands-on surgical procedures [3]. One of the most critical aspects of surgical training, especially in spine surgery, is the development of fine motor skills and tactile intuition. These are honed through realistic haptic feedback, the sensation of resistance and texture when manipulating bone or tissue, such as during drilling or screw placement. While VR simulations offer visual immersion and procedural guidance, they often lack this essential physical resistance, leading to a gap between virtual training and real-world performance. Previous studies have attempted to bridge this gap using force-feedback joysticks or haptic simulators, yet these systems remain costly, technically complex, and often limited in their ability to realistically mimic the biomechanical properties of human bone. This highlights the need for affordable, anatomically accurate physical models that can provide consistent, realistic tactile feedback during practice.

With advancements in 3D printing technology, anatomically accurate and cost-effective physical models have emerged as a viable alternative for surgical training [4]. They can be customized to replicate specific patient pathologies and provide realistic tactile feedback, particularly in procedures involving bone drilling and screw fixation. In this context, 3D-printed spine models serve as an invaluable tool for neurosurgical education, allowing surgeons to practice complex procedures before performing them in real clinical settings.

The lumbar spine is a critical region in spinal surgery, as it bears significant biomechanical loads and is frequently affected by degenerative diseases, trauma, and deformities [5]. Surgical fixation of the lumbar spine, particularly in the region between T12 and the sacrum, requires precise screw placement and stabilization techniques to ensure optimal patient outcomes.

Several challenges arise in training surgeons for lumbar spine fixation [6]. Bone density varies and the vertebral structure changes with age and pathology, requiring different fixation strategies. There is limited access to training resources due to expensive and strict handling protocols of existing cadaveric specimens, which restrict the availability for routine practice. Most important is the need for hands-on experience. Virtual simulations and theoretical knowledge alone are insufficient for mastering surgical techniques such as pedicle screw insertion. A physical 3D-printed spine model with realistic biomechanical properties can help bridge this training gap providing a repeatable, accessible, and cost-effective solution for surgical education.

This study presents the development and validation of a 3D-printed lumbar spine model, designed to replicate the anatomical and biomechanical properties of the L1-sacrum segment. The model is derived from CT data, using segmentation based on Hounsfield units (HU), a scale that quantifies tissue density in medical imaging, to accurately isolate bone structures. Missing anatomical elements, such as intervertebral discs, were reconstructed using Boolean difference operations in 3D modeling software, ensuring realistic anatomical integration.

These design and modeling strategies aim to create a training model that not only looks anatomically accurate but also behaves biomechanically close to human tissue, providing essential haptic feedback during surgical maneuvers such as drilling and pedicle screw placement. By integrating these elements, we propose a cost-effective, customizable, and reusable physical simulator that addresses the limitations of both cadaveric and virtual models. The following sections describe the technical implementation and evaluation of the model during hands-on neurosurgical training, contributing to the advancement of high-fidelity simulation tools in surgical education.

## Technical report

To ensure methodological transparency and reproducibility, the development of the model followed a structured workflow, beginning with data acquisition, followed by segmentation and 3D modeling, and culminating in fabrication, testing, and validation. Each phase incorporated iterative improvements based on practical observations and mechanical testing.

The development of the training model is based on real anatomical structures, extracted from a DICOM image of a 40-year-old male patient’s spine, obtained through a CT scan on a 16-slice multidetector CT scanner (GE BrightSpeed, USA). The imaging data was processed using InVesalius 3D (Centro de Tecnologia da Informação Renato Archer, Campinas, Brazil), following the methodology described in our previous study [7]. The extracted anatomical structure was refined to include vertebrae from L1 to S1, preserving their original spatial orientation with the help of Meshmixer v.3.5.0 (Autodesk, Inc., San Francisco, CA).

**Figure 1:**
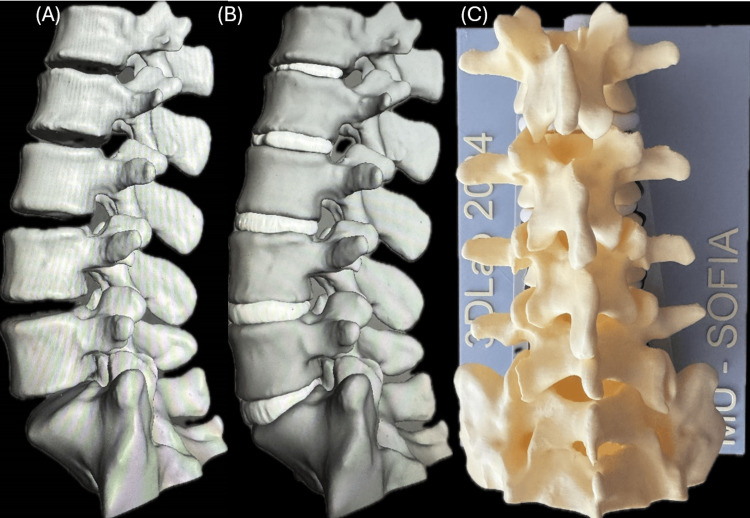
Vertebrae position (A), L1-S1 spinal segment with artificially reconstructed intervertebral discs (B), and the final 3D printed version on the holder (C)

Since the focus was placed primarily on the bone structure, the HU threshold was set to isolate dense cortical and trabecular bone tissue. As a result, intervertebral discs were not visible in their original form, requiring artificial reconstruction (Figure 1).

Intervertebral discs were manually created in Meshmixer to address this limitation by generating cylindrical objects positioned between each adjacent vertebra. The disc dimensions were defined to ensure that their diameter matched or exceeded the curvature of the larger vertebra. At the same time, the height was adjusted to allow adequate contact with both adjacent vertebrae. Each disc was oriented along the axis connecting the centers of the vertebrae as volumetric bodies.

The Boolean difference operation was applied between each vertebra-disc pair to enhance anatomical realism and ensure seamless integration, creating homogeneous contact surfaces between the artificial discs and vertebrae. Further surface refinements were performed using the RobustSmooth tool with the volume brush (strength: 68, size: 30) from the sculpt menu. Special attention was given to the boundary regions between the outer surface of the disc and its contact area with the vertebrae, ensuring a structure that closely resembles real vertebral-disc interactions.

For structural integrity, a 16 mm cylindrical guiding rod was inserted through the center of all vertebrae and intervertebral discs. This ensured that the entire model remained connected while still allowing flexibility in all directions. To facilitate the fabrication process, the guiding rod was subtracted from each vertebra and disc separately using the Boolean difference in Meshmixer.

The primary function of the model is to serve as a training tool for pedicle screw fixation, which requires controlled movement in the transverse plane while limiting motion in the sagittal and frontal planes. To achieve this, the model was embedded in a custom-designed base in the form of a rectangular parallelepiped. To ensure proper spatial orientation and controlled movement, the base featured a negative vertebral column imprint scaled to 105% of the original model size. This enlargement allowed restricted motion within the cavity, simulating realistic biomechanical constraints encountered in clinical scenarios.

The model was produced using FDM (fused deposition modeling) 3D printing (Figure 1C), utilizing a Bambulab X1C printer (Bambu Lab, Austin, TX). Different materials were selected to replicate the biomechanical properties of natural spinal structures. Vertebrae and support base were printed by PLA (polylactic acid, 3Dline, Yambol, Bulgaria) for rigidity and durability. Intervertebral discs and guiding rods, made of PolyFlex TPU95 (Polimaker, Changshu, China), were chosen for their flexibility and elastic properties. The intervertebral discs and guiding rod were printed using a 0.4 mm nozzle with standard settings, including a layer height of 0.2 mm, a wall thickness of 3 perimeters, 3 solid layers on the top and bottom, and 15% infill density with a grid structure. The details were oriented with the transverse plane parallel to the print bed. Standard parallel wall supports were used. The holder was manufactured using the same print parameters.

Initial printing attempts revealed that the external surface of the vertebrae was prone to cracks when subjected to mechanical stress during drilling or pedicle screw placement. This prompted a series of iterative modifications to print settings, including increased wall thickness and layer reinforcement, aimed at improving structural durability without compromising anatomical detail. These refinements were guided by repeated hands-on testing and user feedback from training sessions. This issue prompted an optimization, leading to refined settings of a minimum of 5 perimeters for external walls and 10 solid top and bottom layers, with an outer surface thickness of at least 2 mm to withstand mechanical stress.

**Figure 2:**
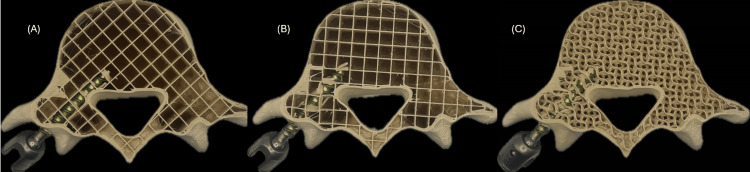
Grid (A and B) and gyroid (C) infill patterns at 15% densities

Another key factor influencing screw anchoring and overall tactile feedback was the internal infill pattern. During initial testing with grid infill, it was observed that the contact surface of the screw was highly dependent on its orientation relative to the print axes. This led to inconsistent resistance during insertion and removal, see Figure 2. A comparative analysis evaluated contact surface integrity between grid and gyroid infill patterns at different densities ranging from 15% to 30% in 5% increments. Results indicated that a 20% gyroid infill provided the best balance between structural integrity and tactile realism, by improving screw anchoring and enhancing the training experience for the user. This comparative evaluation played a crucial role in refining the model’s haptic realism and structural integrity, demonstrating how empirical testing informed design decisions and ensured that the training experience closely resembled real surgical conditions.

**Figure 3:**
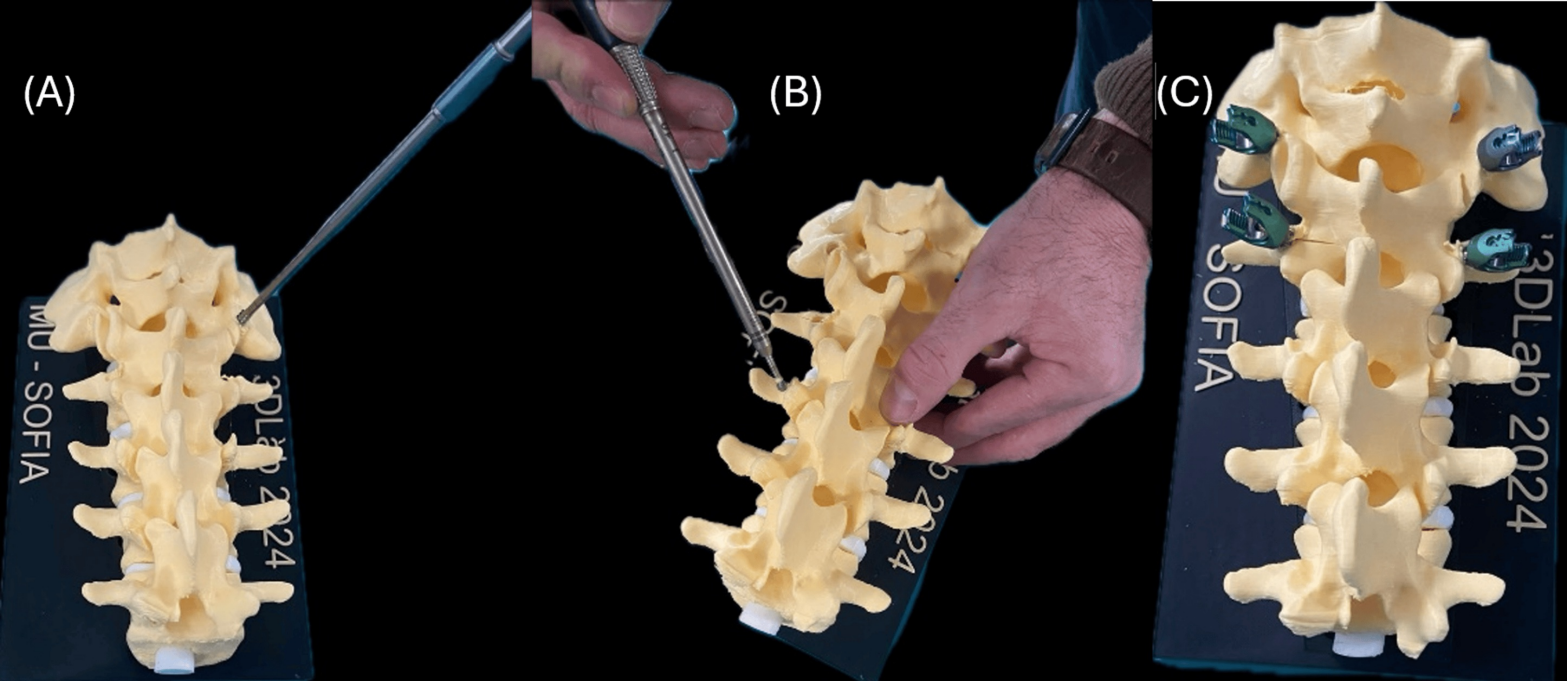
Stages of training: initial hole drilling (A), screw insertion (B), and final positioning of the screws (C)

The training model was tested in hands-on neurosurgical workshops at St. Ivan Rilski University Hospital in Sofia, where it was used for practicing pedicle screw placement with the DePuy EXPEDIUM® 5.5 Spine System, USA. The model’s ability to integrate seamlessly into existing surgical training workflows, through realistic screw insertion, X-ray compatibility, and modular design, further confirms its practical utility. Each design decision, from material selection to geometric layout, was evaluated not only for mechanical feasibility but also for educational value. As shown in Figure 3, the training process includes stepwise execution, from drilling the initial entry point (A), to inserting the pedicle screw (B), and evaluating final positioning (C). This visual sequence reinforces the hands-on applicability of the model in procedural simulation.

**Figure 4:**
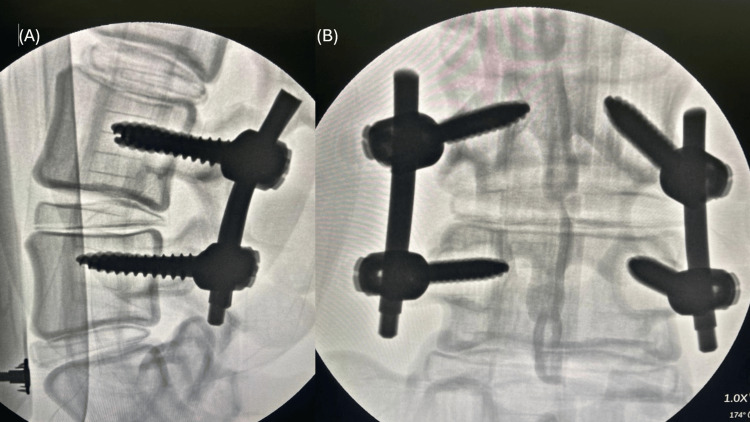
X-ray images of models with implanted screws, assessed for accuracy

The feasibility of the model was evaluated using visual inspection of pedicle screw positioning to determine spatial accuracy and post-procedural X-ray assessment (Siemens Arcadis Orbic 3D, Germany) of models with implanted screws to verify positioning relative to the vertebral structures. The use of radiopaque materials in the model fabrication allowed clear visualization under X-ray, further validating the training process, see Figure 4.

## Discussion

The development of realistic neurosurgical training models has evolved significantly with various approaches offering distinct advantages and limitations. Traditional cadaveric specimens, silicone-based models, and VR simulations each contribute to surgical education in different ways. The proposed 3D-printed lumbar spine model seeks to integrate key advantages of these existing methods while addressing their limitations, offering a cost-effective, customizable, and reproducible training tool for pedicle screw fixation procedures.

Cadaveric specimens remain the gold standard in neurosurgical training due to their high anatomical accuracy and biomechanical realism [8]. They allow for natural haptic feedback, closely mimicking live tissue during surgical interventions. However, their use comes with significant limitations, including limited availability and high costs, as cadaver procurement, storage, and preparation require specialized facilities and adherence to regulatory compliance. Additionally, ethical and legal concerns arise due to the necessity of donor consent and the broader ethical considerations associated with using human specimens. Another major drawback is their non-reusability, as cadaveric specimens cannot be repeatedly utilized for the same procedures once used. While cadaveric models provide the most realistic training experience, their inaccessibility and cost make them impractical for routine training, particularly in institutions with limited resources.

Silicone-based anatomical models offer greater accessibility compared to cadavers and are frequently used for basic surgical training [9]. They are reusable and cost-effective while still providing some degree of haptic realism. Nonetheless, they have notable limitations, including a lack of biomechanical fidelity, as silicone does not replicate the structural composition of cortical and trabecular bone, making it unsuitable for procedures involving drilling and screw fixation. Additionally, their standardized, non-customizable design poses a challenge, as silicone models typically lack patient-specific anatomical variations, unlike DICOM-based 3D-printed models, which limits their educational effectiveness.

Advances in VR technology have introduced highly interactive and customizable training environments. VR-based simulations allow for risk-free procedural practice, providing visual and cognitive reinforcement of surgical techniques. VR models fall short in several critical areas, including the absence of haptic feedback, as they do not provide realistic tactile resistance like physical models, which is essential for training in bone drilling and screw placement. Additionally, their reliance on specialized hardware and high setup costs pose a challenge, as effective VR simulations require advanced equipment and software, making them less accessible for widespread adoption.

The 3D-printed model presented in this study bridges the gap between physical realism and accessibility, offering a cost-effective, patient-specific, and customizable alternative for neurosurgical training. A key requirement for an effective pedicle screw training model is its ability to withstand mechanical stress during drilling and screw placement while providing realistic resistance similar to natural bone. The PLA vertebrae in the proposed model offer sufficient hardness, mimicking cortical bone, while the gyroid infill structure ensures adequate resistance for screw fixation.

While the current 3D-printed model provides an effective and realistic training tool, several areas can be further refined [10,11] to enhance its educational value and biomechanical accuracy. Multi-material printing could be explored by incorporating reinforced PLA composites or variable-density materials to better simulate cortical and trabecular bone structures. Investigating resin-based 3D printing technologies, such as SLA and DLP, may provide higher-resolution anatomical details and improved bone-like textures. Additionally, introducing variable infill densities for different vertebral zones, such as denser cortical regions and more porous trabecular sections, could enhance biomechanical accuracy. Developing removable or replaceable intervertebral discs would allow for varying levels of flexibility depending on the training scenario. 

Furthermore, overlaying augmented reality (AR) guidance onto the 3D-printed model could enhance training precision by combining the benefits of physical realism with digital visualization. AR-based tracking could also assist in real-time assessment of screw trajectory and depth, improving surgical accuracy and feedback. Expanding the model to include pathological conditions such as scoliosis, osteoporosis, and vertebral fractures would provide training opportunities for handling complex spinal deformities and patient-specific cases. Finally, developing a modular system where different spinal regions, such as cervical or thoracic sections, could be interchanged would enable a broader range of training applications.

## Conclusions

Compared to cadaveric, silicone-based VR models, the 3D-printed lumbar spine model offers a cost-effective, anatomically accurate, and customizable solution for surgical training. The study demonstrated that optimized 3D printing parameters, including wall thickness, infill type, and material selection, significantly improve the model’s structural integrity and mechanical reliability during drilling and screw fixation.

The successful implementation of this model in neurosurgical workshops highlights its practical value in medical education. With continued refinement, such models have the potential to become a standardized training tool, bridging the gap between traditional cadaveric methods and modern digital simulations.
